# Correction: Synergies of integrated pest and pollinator management in avocado farming in East Africa: An ex-ante economic analysis

**DOI:** 10.1371/journal.pone.0303547

**Published:** 2024-05-08

**Authors:** Charity Wangithi, Beatrice W. Muriithi, Gracious Diiro, Thomas Dubois, Samira Mohamed, H. Michael G. Lattorff, Benignus V. Ngowi, Elfatih M. Abdel-Rahman, Mariam Adan, Menale Kassie

The sixth author’s name is spelled incorrectly. The correct name is: H. Michael G. Lattorff. The correct citation is: Wangithi C, Muriithi BW, Diiro G, Dubois T, Mohamed S, Lattorff HMG, et al. (2022) Synergies of integrated pest and pollinator management in avocado farming in East Africa: An ex-ante economic analysis. PLoS ONE 17(7): e0271241. https://doi.org/10.1371/journal.pone.0271241.
