# Supplementary material for: Synergies of integrated pest and pollinator management in avocado farming in East Africa: An ex-ante economic analysis
Source: PLoS One. 2022 Jul 25;17(7):e0271241. doi: 10.1371/journal.pone.0271241 (PMC9312383; doi:10.1371/journal.pone.0271241)
Supplement: Attachment — Submitted filename: Review-of-Wangithi_2022_et-al_PLOS_One.pdf [file pone.0271241.s002.pdf]

Review Wangithi et al., PLOS ONE, 2022

General

This is a potential important study on benefits of IPM management and with the inclusion of pollination services the IPPM managements. However, there are some unclarities that makes it hard to evaluate where the assumptions are based on and the definition of some key terms.

Especially, the IPPM needs a clear definition of what is added to the IPM. As is now, it is hard to evaluate, as key terms seems to be intermingled or not well defined.

Introduction

Line 60-64: Would be good to have a definition of what the authors mean by **IPM** and **IPPM** as this can potentially vary between systems and regions. For examples, I would not say adding managed bees to a system makes it a IPPM-system. In comparison, adding bought natural enemies to a crop does not makes the system a IPM-system. Why does the addition of honeybee hives deserves to be called IPPM?

Conceptual models and estimation methods

Line 91: Are you sure that this statement is true? For example, farmers can lack information to base the decision to convert to IPPM. Or there might be a investment cost that is to high for some farmers, even if the practice will generate income in long term.

Line 111-113: Is this not dependent on whether growers have access to markets both locally and export markets and or distributors? So perhaps growers would sell on local markets if they are more accessible or vice versa.

Line 124-127: Where does this expectation come from? Do you have any references? I would *not* expect the effect to be direct, especially not for biological control, as it can take some time to increase populations of natural enemies and pollinators. If you support pollination with managed pollinators this will be quicker of course.

Line 161: Why is the equations jump from 7 to 9? Is there a nr 8 that is missing or is that just a skip of numbers?

Data sources, parameter estimation and assumptions

Line 183-186: Here you state that there are three options, *IPM*, *pollinator supplementation* and *IPPM*. However in line 60-62 you state that the IPPM-package integrates IPM with manage pollinators.

So if you chose IPPM does it include both IPM and manage pollinators? Could you clarify this. Again, what is the definition of IPPM here?

Line 237-239: It seems you take the 20.7% value from the Sagwe et al. 2021 paper or did you calculate the current pollination deficit? Is taking figures from Sagwe et al., 2021 reliable as you have farmers in both Kenya and Tanzania? Do you include only smallholder farmers?

Results and Discussion

Line 303?: ***Line numbering stopped so I had to guess*** The note March 2022ˆ1 should be used as a regular reference instead.

Mid sentences, sec. 4.2: Now you equal IPPM with beekeeping again, but wasn't managed bees a separate thing from IPPM?

Last sentence, Sec. 4.2: How does beekeeping increase pollinator diversity?

In general I think you need to discuss the sensitivity analysis a bit more as it was very sensitive to the yield gain. It seems very unsure how big that will be as it is currently based on a secondary report from small scale farmers in one of the countries.

Conclusion

I think you would benefit by including your mayor findings and how it impact farmers in the end of the conclusion as a final statement.

Tables

Table 2 In the *Note* it says PS, CS, PS, I guess the last PS should be *TS*.

Table 2 and 3 If CS is always zero, is it not sufficient to say that in the Table text and then remove the CS and TS columns?
