# Supplementary material for: Synergies of integrated pest and pollinator management in avocado farming in East Africa: An ex-ante economic analysis
Source: PLoS One. 2022 Jul 25;17(7):e0271241. doi: 10.1371/journal.pone.0271241 (PMC9312383; doi:10.1371/journal.pone.0271241)
Supplement: Attachment — Submitted filename: Comments to authors.docx [file pone.0271241.s003.docx]

**Comments to authors**

The manuscript is valuable and efficient contribution towards the implementation of novel strategies of Integrating Pests and Pollinator Management. However some suggestions are given below for miner revision.

Title

Is good but needs miner rephrasing. Reduce the use of (in)

Abstract

This portion may be good if you add some methodology at line 22 before results. Results may also be elaborated in numerical terms as you have done for cost benefit ratios etc. At the last part of your abstract must indicate future research corridor missing in your research plan.

**Keywords**

Good

**Introduction**

At line 73 you must add review about the similar studies already done on any other such crop and their outcome in the form of mini review. Then you must come to your studies.

Line 80-87 seems parts of your results and discussion. These lines may be replaced with the objectives of your studies.

**Material and methods**

I am more concerned with your methodology as it lacks an integrated methodology you opted. Although you have given concepts, theories before each methodology for subject specialists but for common readers you must elaborate your methodology in a simple form step by step. You may give a schematic diagram of your methodology what were your first step etc. How you started and then ended give step by step in more simpler and understandable way. How you used different formulas and then applied model and how you validated the results of your model. All this can be done with the help of a schematic diagram showing every step and model application procedure.

Results

Before recommendations results must be expressed in a consolidated form.

Discussion must be with citations of similar studies on any other crop if any.

**References**

References must be according to the format of the journal. This part need the attention of the authors

**General Comments**

Manuscript is well written. Authors must follow journals format and miner grammatical and language corrections.
