# Supplementary material for: Synergies of integrated pest and pollinator management in avocado farming in East Africa: An ex-ante economic analysis
Source: PLoS One. 2022 Jul 25;17(7):e0271241. doi: 10.1371/journal.pone.0271241 (PMC9312383; doi:10.1371/journal.pone.0271241)
Supplement: Attachment — Submitted filename: Comments of Reviewer No 3.docx [file pone.0271241.s004.docx]

**Comments of Reviewer No. 3**

Article lacks some important details to be fully understand. In addition, it is not well highlighted the importance of this study, and how the finds could be used in future. Please take into account that many sentences need to be rephrased, in my opinion. Moreover, the results should be further developed in the discussion and improve the discussion portion. Some suggested changes as an example are in the comments portion to revise and improve the manuscript. There are many sentences throughout the manuscript which is hard to understand. The present form of draft required a lot of corrections. Please find the comments and suggested corrections. Complete editing corrections, journal-style format, use of abbreviation and missing information should be maintained.
Title: Revise the title of the paper
Abstract: Revision of the abstract is required.
Line 20-22: The authors mentioned “aims to evaluate the potential impact of IPPM elements…? which elements?
Line 22-23: Our results show that the potential economic gain from the adoption of IPPM? What kind of adoption? especially in pest management?
Line 26-29: Restructure the sentence
Rewrite the abstract portion as this is not understandable. The English writing is so confusing to read, it is therefore suggested to rewrite the abstract. English words, vocabulary, journal-style format, word spacing and use correct abbreviations are recommended
Introduction:
Line 44: Restructure the sentence
Line 52-54: Add some relevant references.
Line 69-71: Previous studies have mainly evaluated the economic benefits of IPM technologies and beekeeping in isolation: Add some references to support the statement? which studies?
Line 80-84: The results show significant potential gains……….persons per year out of poverty in Kenya and Tanzania, respectively. This is result portion; add this portion in the results of the study.
Line84-87, 115-116: Restructure the sentence. These sentences are more confusing to understand
Line 131-151: mention the reference of the formulas used in the draft
Line 179-181: These include Murang’a County in Kenya and Kilimanjaro 180 Region in Tanzania…… accounting 181 for 46.9% of the total value of avocado production in the country” Restructure the sentence as this is confusing.
What kind of insects and diseases are present in the avocado crop and what other methods farmer utilized to avoid the chemical control and their economic impact?
Put the survey files in supplementary data as annexures
Line 214-216: Restructure the sentence as this is confusing to understand.
Data sources, parameter estimation, and assumptions” The draft under this sub-headline is too lengthy. Please concise the draft of this subheading
Please add latest reference. Please also see the author’s guidelines of the journal about reference writing. Please mention the research question of why you did this study?
Conceptual model and estimation methods/Materials and Methods:
MM portion needs revision. The sentences are written carelessly; please restructure the sentences and editing used during the study.
Data analysis. Restructure the sentences and add references utilized during the analysis
Results:
The economic analysis of integrating IPM with pollinator supplementation over a 15-year …….which 15 years restructure the sentence as this is confusing (mention the years e.g 2022-2037).
Line 254-260: The authors mentioned the economic benefits from the studied assumptions however they didn’t mention the alternative methods to control insect pests and their economic impact. Please mention this in discussion portion.
The economic comparison of the other alternative crop protection methods to reduce the chemical control in the studied crop area is missing in the discussion portion?
The results portion needs revision. The result portion needs restructuring of the sentences as in the present form the English of the results is not understandable. It is therefore recommended that the authors read to improve the sentences after suggested corrections and before submission.
Discussion:
The findings suggest that the adoption of sustainable pest management strategies and enhancing pollinators' biodiversity through beekeeping has an instrumental role in economic growth and against poverty reduction in Kenya and Tanzania………. What kind of sustainable PMSs?

The discussion portion is very weak and without the discussion of the study conducted. Please add the necessary discussion in the manuscript with the latest reference of the study conducted. Add some latest references in the discussion portion.
Groups of references can be listed either first alphabetically, then chronologically, or vice versa. But you need to be consistent throughout the text. Please correct this in the whole manuscript.

Conclusion:
Conclusion and policy recommendations portion is too lengthy draft and these portions should be written separately in more concise form without the repetition of the information. The conclusion portion is not fine in the present form. Please restructure the conclusion portion by adding the relevant conclusions, and also add the future implication of the present study. Please rewrite in a more concise form.
Figures:
Please also take care of the formatting utilized; there should be uniform formatting throughout the figures. Please also correct the titles by adding the used abbreviations of the figures and formatting according to the author’s instruction of the journal
Tables:
Please correct the titles numbers of the tables in the manuscript.
References: Follow the journal style formatting?
Please add the latest references in the whole manuscript and in literature cited portion
Please double-check for typos and inconsistencies in Journal style/formatting as, among others, missing italics, missing information
